# Optimal Pipe Size Design for Looped Irrigation Water Supply System Using Harmony Search: Saemangeum Project Area

**DOI:** 10.1155/2015/651763

**Published:** 2015-03-19

**Authors:** Do Guen Yoo, Ho Min Lee, Ali Sadollah, Joong Hoon Kim

**Affiliations:** School of Civil, Environmental and Architectural Engineering, Korea University, Seoul 136-713, Republic of Korea

## Abstract

Water supply systems are mainly classified into branched and looped network systems. The main difference between these two systems is that, in a branched network system, the flow within each pipe is a known value, whereas in a looped network system, the flow in each pipe is considered an unknown value. Therefore, an analysis of a looped network system is a more complex task. This study aims to develop a technique for estimating the optimal pipe diameter for a looped agricultural irrigation water supply system using a harmony search algorithm, which is an optimization technique. This study mainly serves two purposes. The first is to develop an algorithm and a program for estimating a cost-effective pipe diameter for agricultural irrigation water supply systems using optimization techniques. The second is to validate the developed program by applying the proposed optimized cost-effective pipe diameter to an actual study region (Saemangeum project area, zone 6). The results suggest that the optimal design program, which applies an optimization theory and enhances user convenience, can be effectively applied for the real systems of a looped agricultural irrigation water supply.

## 1. Introduction

Water supply systems are mainly classified into branched and looped network systems. The main difference between the two is that, in a branched network system, the flow within each pipe is a known value, whereas in a looped network system, the flow within each pipe is considered an unknown value. Therefore, an analysis of a looped network system can be a more complex endeavor.

Water supply systems form part of a larger social infrastructure of an industrial society; their objective is the effective supply of water from a water source to an area in demand. The analysis of a water supply system can be one of the more complex mathematical problems. A significant fraction of the entire set of equations consists of nonlinear equations, and a large number of these equations must be solved simultaneously.

This process requires sufficient consideration of the law of conservation of energy and a continuity equation of mass. In this regard, over the past few decades, many methods have been developed to analyze water supply systems and perform hydraulic simulations of their steady state conditions. Commercial hydraulic analysis programs such as EPANET [1] and WaterGEMS [2] have been developed to analyze the hydraulic simulations of large water supply systems, an achievement that could not even be dreamed of in past years.

The development of such models has played an important role in the design and operation of water supply systems. However, problems related to the selection of the pipe diameter for configuring low-cost water supply systems have emerged as important issues that need to be resolved.

In recent years, many optimization methods have been used for the design of low-cost water supply systems. The process for obtaining an optimal water supply system and pipe diameter is considered important because it helps in determining the final operational costs. However, because the aforementioned problems are extremely complex, they are constrained by the types of methods selected for defining the problems, as well as by analysis methods; thus far, only a minimization of the construction costs has been experimentally applied [3–6].

If the structure of a water supply system and its constraints (water pressure and velocity) are known, the optimal design of the water supply system can be expressed in terms of the selection of a pipe diameter that minimizes the total cost. The mathematical optimization methods described earlier can easily be used to find the optimal solutions in small systems within an ideal environment.

However, if existing mathematical optimization methods are applied to actual civil engineering problems, the limitations of these methods are revealed. For example, in linear programming, because all functions applied to such problems are linear, simplified assumptions lower the accuracy of the final solution. On the other hand, in dynamic programming, too many combinations have to be considered to obtain the optimal solutions, thereby requiring a considerable amount of computational effort and storage space.

In nonlinear programming, if the initial solution is not located at a good position within the solution zone, the global optimum is unobtainable, and the initial solution cannot escape from the local optima. To overcome these disadvantages, during the last 20 years, researchers have attempted to apply new approaches to optimization technologies that do not use an existing mathematical methodology.

Rather than relying completely on conventional differential derivatives, the technologies currently being developed apply a natural evolution phenomenon, that is, the principle of the survival of the fittest, and artificial imitations of this phenomena in the optimal system design; these have yielded better results than those obtained using an existing optimal mathematical design.

Such nature-inspired optimization algorithms are called metaheuristic algorithms. Some examples of such algorithms include a Genetic Algorithm (GA), Simulated Annealing (SA), Tabu Search (TS), Ant Colony Optimization (ACO), Harmony Search (HS), and Particle Swarm Optimization (PSO). Reca et al. [7], Monem and Namdarian [8], da Conceição Cunha and Ribeiro [9], Zecchin et al. [10], Geem et al. [11], and Montalvo et al. [12] have conducted studies on the optimal design of water supply systems using the GA, SA, TS, ACO, HS, and PSO algorithms, respectively. In recent years, hybrid versions of existing algorithms and new algorithms have been also developed such as Genetic Heritage Evolution by Stochastic Transmission (GHEST, [13]), NLP-Differential Evolution algorithm (Combined NLP-DE, [14]), Hybrid Particle Swarm Optimization and Differential Evolution (Hybrid PSO-DE, [15]), and Charged System Search algorithm (CSS, [16]).

However, most of these studies have disadvantages in that they were applied to small benchmark problems and were not reflected in the actual plans [17]. The present study aims to develop an optimal pipe diameter estimation technique of an actual agricultural looped irrigation water supply system using an HS algorithm. This study has two main purposes. The first is to develop an economic pipe diameter estimation algorithm and program using the optimization techniques for agricultural irrigation water supply systems. The second is to validate the developed program by applying the proposed optimized economic pipe diameter to an actual target region (Saemangeum business area, zone 6).

## 2. Saemangeum Project

The Saemangeum Project is a reclamation project intended to create land from a mud flat and sea waters along the western coast of South Korea by constructing a 33.9 km long seawall. Under the Saemangeum Project, which was started on November 16, 1991, the construction of a cofferdam was completed on April 21, 2006, and the reinforcement and embankment projects were completed on April 27, 2010.

The Saemangeum seawall is listed in the Guinness Book of World Records as the longest seawall on record and is 1.4 km longer than the Zuider seawall (32.5 km) of the Netherlands, which was earlier regarded to be the longest. The construction of the seawall has resulted in the reclamation of a new region with an area of 401 km^2^, of which land and a fresh water lake account for 283 km^2^ and 118 km^2^, respectively.

**Figure 1:**
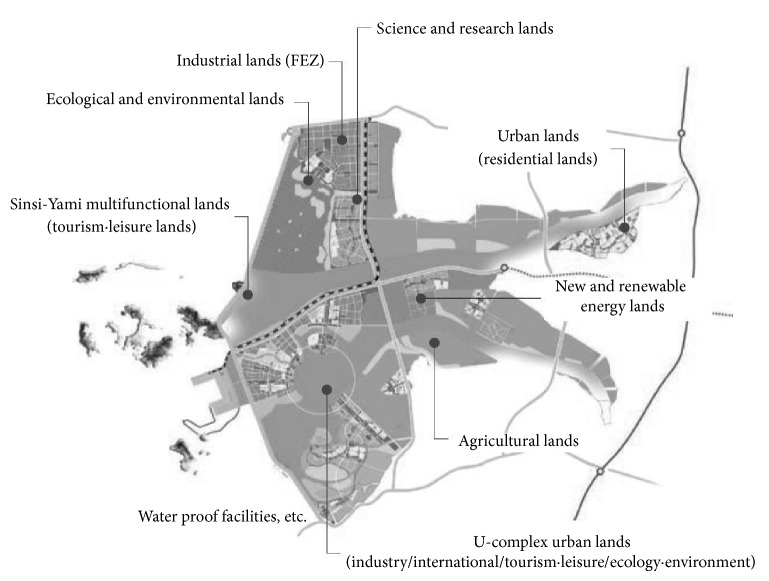
Comprehensive Saemangeum development plan.

Upon completion of the seawall construction, the South Korean Government newly formulated its Comprehensive Saemangeum Development Plan. According to this plan, the reclaimed land is to be developed as the central agricultural and economic sector of Northeast Asia, with the reclaimed land mainly divided into nine areas, as shown in Figure 1 and Table 1.

Agricultural lands account for the largest share (30.3%) among the divided areas. These agricultural lands are used for ensuring national competitiveness, producing high-value-added agricultural products, and developing food-industry facilities through mixed environment-friendly agriculture and ecological crop cultivation. Traditionally, agricultural irrigation water supply systems have been designed as branched water supply networks, which incur less initial costs. However, such networks are disadvantageous in that they do not ensure the reliability of the water supply.

In recent years, new cultivation methods such as greenhouse crop cultivation, high-value-added crop cultivation, and perennial cultivation have been adopted. Accordingly, agricultural irrigation water supply systems also need to provide a stable and reliable water supply, as achieved by urban water supply systems.

## 3. Model Development and Methodology

### 3.1. Harmony Search Algorithm

The HS algorithm proposed by Geem et al. [11] is an optimization technique used in pipe design. HS is a solution-finding technique that considers an optimal solution in engineering to correspond to an optimal sound in music. Generally, heuristic search methods involve the observation of natural phenomena, but the HS method is an algorithm based on the artificial phenomenon of harmony.

When sounds are produced by various sources, they together create a single harmony. Some of these created harmonies sound pleasant, whereas others sound dissonant. Eventually, the discordant harmonies disappear through practice, and among the more appropriate harmonies (local optimum), those that are aesthetically the most beautiful (global optimum) are achieved.

In other words, the HS algorithm considers an optimal solution to be an optimal harmony found through practice. The principle of the HS algorithm can be explained in detail by first comparing how music improvisation and optimization calculations correspond to each other.

**Figure 2:**
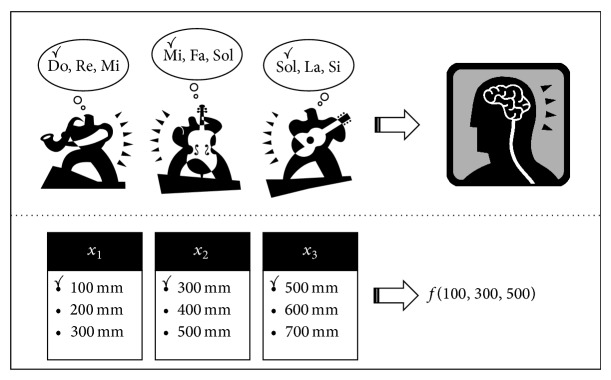
Concepts of a harmony search.

Improvisation is the spontaneous creation of notes by performers without relying on sheet music (score). The ability of the performers improves the more they perform together, and ultimately a top-level harmony is created. In such an improvisation, each performer (e.g., a saxophonist, guitarist, and double bass player, as shown in Figure 2) can be referred to as a decision variable or design variable (*χ*_1_, *χ*_2_, and *χ*_3_ in Figure 2).

The musical range of each instrument (in the case of the saxophonist, e.g., one of the notes among Do, Re, and Mi, can be created) made by the corresponding performer can be referred to as the range of each variable (in the case of *χ*_1_ in Figure 2, its pipe diameter may be 100, 200, or 300 mm). Moreover, when each performer plays a different note, the harmony they create (e.g., the harmony in the figure) (i.e., saxophone, Do; double bass, Mi; and guitar, Sol) corresponds to the overall solution vector obtained (the solution vector for Figure 2 is *χ*_1_ = 100 mm, *χ*_2_ = 300 mm, and *χ*_3_ = 500 mm) by substituting the value of each variable.

Whether the harmony played at a point in time is of high quality is judged aesthetically by the performers or audience through auditory stimuli. If the harmony is very pleasant for the performers or audience, it will often be replayed in the memories. Likewise, during an optimization, whether a solution vector is good or bad can be determined by substituting the vector in an objective function; if this yields a better functional value than the existing one, the solution vector will be preserved.

Moreover, in an improvisation, as the performance is repeated, better harmonies are created, and ultimately, a high level of ability is reached; likewise, in an optimization operation, as additional iterations are carried out, better functional values are increasingly developed, and ultimately, the optimum value is obtained.

The harmony memory (HM), harmony memory considering rate (HMCR), and pitch adjustment rate (PAR) are important factors in the HS method for finding an optimal solution. First, each musical performer should have a memory space to preserve a good harmony; before starting the important process of the HS algorithm, a harmony memory space is created by consolidating existing memory spaces.

This is called the HM, and the maximum number of harmonies that can be stored in this storage space is called the harmony memory size (HMS). Next, to produce better solutions from the harmony storage space, which is initially filled by as many random vectors as the HMS, the HS algorithm employs three types of operators.

#### 3.1.1. Random Selection

In the random selection technique, the value of a variable is randomly selected from all values of the playable note range. If *K* is the total number of all possible variable values, one of them is randomly selected, and the probability of this technique being adopted is 1-HMCR.

#### 3.1.2. Memory Consideration

The memory consideration technique picks the value of a variable from the existing high-quality notes. In other words, a single value is picked from all values possessed by a variable within the storage space. Its probability is HMCR, and although it can have a value between 0 and 1, a value between 0.7 and 0.95 is usually used; nevertheless, the value is changeable.

#### 3.1.3. Pitch Adjustment

For a pitch adjustment, a note obtained through a memory recall technique is considered a basic note, and its pitch is trimmed by adjusting the note based on the surrounding upper and lower notes. In an actual calculation, when a single value is obtained using a memory recall technique, it is adjusted by a one-step higher or lower value. The PAR is the probability of this technique actually being applied, and it can attain a value between 0 and 1. Generally, the PAR has a value of around 0.01 to 0.3, but this can vary.  Pseudocode 1 shows the pseudocode of the HS algorithm.

### 3.2. Objective Function

An objective function minimizes the design cost of an irrigation system. The algorithm developed in the present study was applied to an optimization; the construction costs, pipe material costs, and maintenance costs are considered as the design costs according to the pipe diameter. Therefore, the equation for the objective function is as follows:

(1) $$\begin{array}{c} \mathrm{Min}\cdot \;\text{Cost}=\overset{N}{\underset{i=1}{\sum }}\left(C_{C}\left(D_{i}\right)+C_{M}\left(D_{i}\right)+C_{P}\left(D_{i}\right)\right)L_{i}, \end{array}$$

where *C*_*C*_(*D*_*i*_) is cost function (construction cost) per unit length (m) for each pipe diameter, *C*_*M*_(*D*_*i*_) is cost function (maintenance cost) per unit length (m) for each pipe diameter, *C*_*P*_(*D*_*i*_) is cost function (pipe material cost) per unit length (m) for each pipe diameter, *L*_*i*_ is length of the pipe (m), *D*_*i*_ is pipe diameter (mm), and *N* is total number of pipes.

Hydraulic constraint equations are considered in optimization problems. Therefore, a penalty function method is introduced to convert the optimization problem subject to constraint conditions into an optimization that is free from the constraint conditions. The final objective function, which is applied using a penalty function, can be defined as follows:

(2) Min⁡· Cost=∑i=1NCCDi+CMDi+CPDiLi +∑j=1MPjhj−hmin⁡ or max⁡ +∑i=1NPivi−vmin⁡ or max⁡,

where *h*_*j*_ is pressure head of each node (m), *h*_min⁡_ is minimum pressure head (m), *h*_max⁡_ is maximum pressure head (m), *v*_*i*_ is velocity of each pipe (m/s), *v*_min⁡_ is minimum pipe velocity (m/s), *v*_max⁡_ is maximum pipe velocity (m/s), *P*_*j*_, *P*_*i*_ are penalty functions with regard to the pressure and pipe velocity, and *M* is total number of nodes.

The above penalty function is applied only when the pressure of each node and the velocity of the pipe exceed either the minimum or maximum value; the equation below represents the penalty function equation applied to the present model. In the target water supply system, the minimum and maximum nodal pressures were set to 10 and 35 m, respectively, and the minimum and maximum pipe velocities were set to 0.01 and 2.5 m/s, respectively:

(3) Pj=αhj−hmin⁡  or  hmax⁡−hj+β,Pi=αvi−vmin⁡  or  vmax⁡−vi+β,

where *α*, *β* are penalty constants.

When running an optimization model, if the pressure head of each node and the velocity of the pipe do not satisfy the minimum and maximum values, which are the design conditions, the penalty cost is increased by assigning a significantly greater value to *α* so that the solution will not be selected. To prepare for a case in which the pressure head and pipe velocity fall short of the design conditions by a small margin, a model that largely satisfies all of the design conditions was implemented by assigning a large value to *β*. A trial-and-error analysis was conducted using the *α* and *β* values for Saemangeum, which is the target area of the present project. The results indicate that an effective optimal design is possible when *α* and *β* are assigned values of 10,000,000 and 100,000,000, respectively. But, detailed studies about constraint handling techniques and determination of their parameters should be tackled to improve model efficiency and reliability in future.

## 4. Saemangeum Water Supply Network Application and Results

### 4.1. Target Water Supply Network

**Figure 3:**
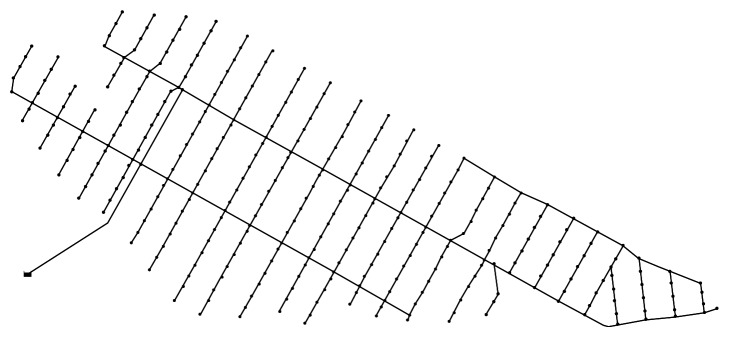
A looped water supply network applied to the target zone of Saemangeum.

In the present study, proposal data on the loop-type design of the six zones of Saemangeum were obtained and applied to one of the zones. A diagram of the corresponding water supply network is shown in Figure 3. The target water supply network comprises 356 pipelines, and as mentioned earlier, some of the network consists of a circuit-type water supply.

The data on the cost incurred per unit of pipe length for the different diameter pipes used in this study are listed in Table 2. For optimization, 18 types of commercial pipes with different diameters were considered. Data on the construction and pipe material costs corresponding to the different pipe diameters were obtained from the “Water Facilities Construction Cost Estimation Report” from K-water [18], which provides estimated data on the construction costs for different steel pipe diameters. The task of optimization was carried out on Intel(R) Core(TM) i5-3570 CPU at 3.4 GHz with 4 GB RAM. EPANET [1] was used as a hydraulic analysis program.

### 4.2. Parameter Settings

The number of decision variables, which should be determined through optimization, is 356 because there are 356 pipelines in the target water supply network. As indicated in Table 2, 18 pipe diameters were considered for the target water supply network. Hence, the number of possible solutions that can be considered during the design period is infinite, as mentioned in Table 3.

The parameters applied in the present program for the Saemangeum target water supply network are listed in Table 4. The size of the harmony memory (HMS), the value of the HMCR parameter, and the value of PAR were set to 30, 0.97, and 0.01, respectively.

These values, which correspond to the optimum results, are adjusted; therefore, the convergence time and efficiency of the optimal solution vary. However, when there are many decision variables, and in such a case, if large HMCR and small PAR values are used, the efficiency of the optimization generally increases.

### 4.3. The Economic Feasibility of the Initial Design and Hydraulic Analysis Evaluation

To compare and evaluate the optimization results of the pipe diameters for the initial design, the cost results and hydraulic analysis results of the initial design were first reviewed according to Pseudocode 1; the results of this review are listed in Tables 5 and 6. The equalization of the nodal heads and the economical velocity corresponding to each pipe diameter are generally used as the factors in evaluating the mathematical stability of an irrigation system.

The minimum nodal pressure head is mostly stable at a value greater than 10 m. In the present initial design, a looped network irrigation system is implemented by installing an additional pipeline to a branched network system. In this case, the supply path up to the demand node is determined to be a branched network, that is, only a single type.

However, in the initial design, because various supply paths are possible, the head loss is slight, and a water supply is possible through the hydraulically satisfied supply paths, a system that is more hydraulically stable than a branched network system that can be implemented. Thus, because various supply paths are possible in a looped irrigation water supply system, a looped system provides a better water supply than a branched system during abnormal operating conditions such as during an irrigation path failure or closure.

### 4.4. Optimal Pipe Diameter Design Results

**Figure 4:**
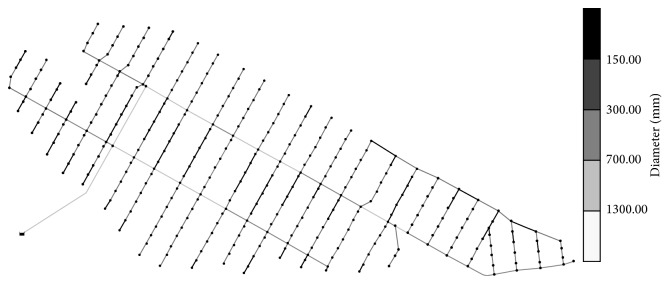
Pipe diameter optimization results for the six zones of the Saemangeum water supply network.

The pipe diameter was optimized by considering the pressure and pipe velocity constraint conditions and the HS parameters, which were explained earlier in this study. The optimization results from a cost-effective pipe diameter are shown in Figure 4.

The statistical values of the nodal pressure head and pipeline velocities, which are the results of a hydraulic analysis based on cost-effective pipe diameter and the optimal cost results, are shown in Tables 5 and 6. Overall, the pressure head and pipe velocities were confirmed to be stable, and a comparison based on the hydraulic stability and economic feasibility of the initial design was conducted.

The application results indicate that the cost reduction rate of the optimal design was considerably greater (9.08%) than that of the initial design. These results were further analyzed from the viewpoint of current practices that do not employ optimization techniques; this analysis indicates that even without using any optimization technique, branched network systems that do not significantly differ from the optimal designs can be created using the current techniques.

However, in the case of a looped network system, such as the water supply network applied in this study, the differences in the results were significant; therefore, it is necessary to determine an cost-effective pipe diameter for the optimization technique based on the results obtained when employing current practices. The hydraulic analysis results indicate that the minimum pressure head (more than 10 m) was mostly satisfied, as observed in the initial design. Furthermore, the statistical values of the nodal pressure head and pipe velocity indicate that the minimum pressure head, allowable pipe velocity, and average pipe velocity all satisfy the economical pipe velocity requirements.

## 5. Differences from Other Existing Plans

**Figure 5:**
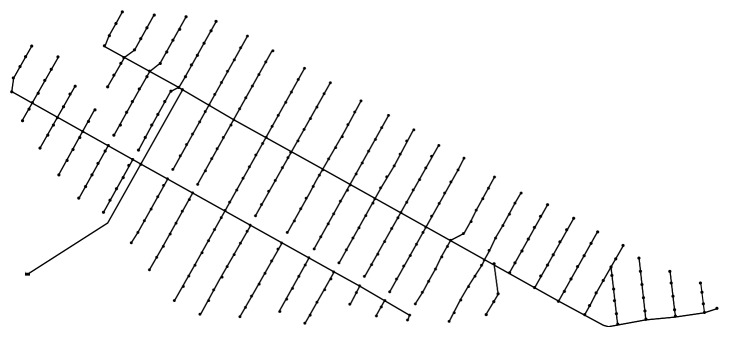
Branch-type system.

**Figure 6:**
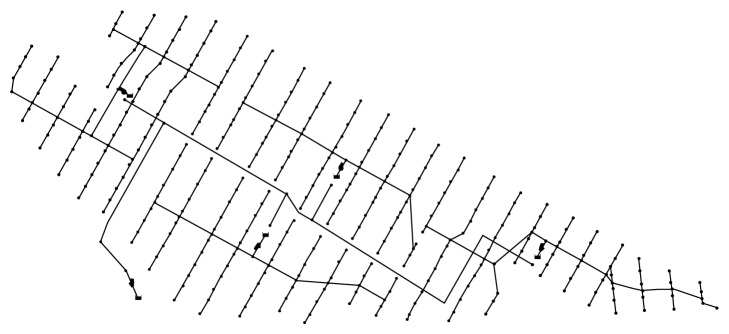
Pump-type system.

In the present study, optimal design reviews of two other design plans in addition to the proposed looped network design plan were conducted. These two design plans are of a branch type and a pump type, as shown in Figures 5 and 6, respectively.

The branch-type water supply network comprises 335 pipelines, with a total length of 37.88 km. The pump-type water supply network comprises 345 pipelines; for the water supplied by the pumping of this irrigation network, the entire area encompassing the six zones was reclassified into four new areas. The total length of the pipelines is approximately 41.39 km.

To compare and evaluate the estimation results for the optimal pipe diameter of the three water supply network systems, that is, the loop type (plan 1), branch type (plan 2), and pump type (plan 3), the cost results according to the final optimum pipe diameter and the pipe diameters of the initial plan of each of the three networks are listed in Table 7.

The results indicate that the cost of applying the optimal design was at a minimum for plan 2 and at a maximum for plan 3. This is similar to the trends found in the initial plan. However, an examination of the varying cost rate shows that the cost reduction of the optimal design for plan 2 was 4.19% less than that of the initial plan. On the other hand, the cost increased by 0.72% for plan 3, whereas in the case of plan 1, the cost reduction rate was very high (9.08%). The results for plan 1 show that the reduction rate between the optimal cost and the total length of the pipes is inversely proportional when the pressure head and velocity conditions remain constant. Moreover, a looped irrigation system has many nodes and pipes, which vary hydraulically because pipes of different diameters are used in a pipe system; this proves that it is difficult to design a looped irrigation system economically without using an optimization technique.

These results are attributed to the fact that the self-nodal pressure head of the initial version of plan 1 is relatively greater than that of the initial version of plan 2. However, from the viewpoint of current practices, which do not employ optimization techniques, branch-type systems such as plans 2 and 3, which do not differ greatly from optimal systems, can be designed by applying current techniques. In the case of a looped network system such as plan 1, the differences between the results corresponding to the initial and optimal designs were considerable. Therefore, based on the results from current practices, it is necessary to determine a cost-effective pipe diameter using an optimization technique.

The results of a hydraulic analysis in which the optimal pipe diameters for plans 1, 2, and 3 were considered are shown in Table 8. The statistical values of the nodal pressure head and pipe velocity indicate that the minimum pressure head, allowable pipe velocity, and average pipe velocity for all three plans satisfy the economical pipe velocity requirements. An examination of the nodal pressure head confirms that the minimum pressure head (10 m) is mostly stable in plans 1 and 2, as is the case of the initial plan. In the case of plan 3, the minimum pressure for the initial plan was very low (0.5 m); however, the cost increases if the minimum pressure of the initial plan (0.5 m) exceeds the minimum pressure standards (10 m) during the optimal design process.

A comparison of the three optimal design types shows that plan 2 (branch type) is the most economic optimal design based only on the criterion of minimum costs. However, because plan 2 does not differ greatly from plan 1 in terms of costs, it is necessary to derive the final design results by considering the hydraulic and maintenance aspects. Plan 1 is a case in which a looped network irrigation system is implemented by installing additional pipelines to plan 2, which is a branched system.

If the pipelines supplied up to the demand node correspond to plan 2 (branch network type), the supply path is determined to be of only one type. However, in the case of plan 1, many supply paths are present; the water supply is made possible through the supply paths, which are hydraulically satisfactory. Therefore, plan 1, which is a more hydraulically stable system than plan 2, can be implemented.

Thus, the supply of a looped irrigation water supply system during abnormal situations such as an irrigation path failure or closure is better than that of a branched irrigation water supply system because the former has various supply paths. Unlike plans 1 and 2, plan 3 was designed by reclassifying the target pipeline system into four hydraulically independent sections, and water was supplied to each section through pumping heads. By dividing the target pipeline system into four hydraulically independent sections, the fluctuations in the water quantities by each area can be more effectively and reasonably handled, and plan 3 can respond to future pipeline maintenance and expansion plans. However, the increased use of pumps can cause maintenance difficulties and an increase in maintenance costs.

## 6. Conclusions

In the present study, the HS algorithm, which is one of the latest optimization techniques, was introduced in the design of an agricultural irrigation system, and a corresponding program was developed. The developed program was applied to the actual target area (Saemangeum business area, zone 6), and the results were presented in this paper. Currently used methods have disadvantages in that the pipe diameter has to be adjusted through a hydraulic calculation of the given water supply network, and this process has to be repeated until satisfactory results are obtained. Unlike this calculation method, the model presented herein yields results that automatically meet the hydraulic conditions through the combined use of the HS algorithm and a hydraulic analysis. Hence, a comparative analysis is simple and effective. The results obtained by applying this method to an actual large-scale water supply network are better than those obtained using existing mathematical algorithms even after considering the nonlinearity, which is inevitable during the analysis. The calculation results of the optimal construction costs and the pipe diameter when applying the proposed model to the actual target region (Saemangeum business area zone 6) indicate that the optimal design results obtained using HS yield much better results (9%) in terms of cost than those of the presently utilized economic pipe diameter calculation techniques. In particular, the optimization technique was found to be more necessary in the optimal design of a looped network irrigation system than for a branched network irrigation system. Furthermore, an examination of the hydrological factors of a pipeline system in which cost-effective pipe diameters were applied showed that based on the statistical values of the head and pipe velocity, the minimum pressure head, the allowable pipe velocity, and the average pipe velocity all satisfy the requirements of an economical pipe velocity. Therefore, if the benefits of the proposed model are proven through application in future systems, it will show the model to be a useful decision-making tool for designing looped network water supply systems.

## Figures and Tables

**Pseudocode 1:**
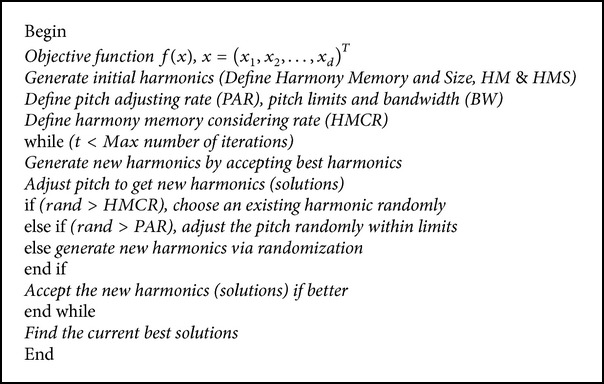
Pseudocode of HS.

**Table 1:** Land use divisions according to the comprehensive Saemangeum development plan.

Division and facilities	Ratio (%)	Area (km^2^)
Agricultural lands	30.3	85.7
U-complex urban lands	23.8	67.3
Industrial lands (free economic zone (FEZ))	6.6	18.7
Science and research lands	8.1	22.9
New and renewable energy lands	7.2	20.4
Urban lands	5.2	14.7
Sinsi-Yami multifunctional lands	0.7	2.0
Ecological and environmental lands	15.0	42.4
Water proof facilities and so forth	3.1	8.8

Total	100.0	282.9

**Table 2:** Cost data corresponding to different pipe diameters for Saemangeum.

Pipe diameter (mm)	Cost (*₩*/m)		
	Construction costs	Material costs	Maintenance costs
80	65,000	15,000	6,500
100	65,999	27,583	6,600
150	76,410	40,686	7,641
200	86,028	58,716	8,603
250	96,135	81,160	9,614
300	105,325	103,231	10,533
350	113,818	125,107	11,382
400	126,797	148,836	12,680
450	136,250	155,522	13,625
500	147,792	181,823	14,779
600	171,991	211,396	17,199
700	211,413	273,528	21,141
800	307,640	339,740	30,764
900	359,048	384,619	35,905
1,000	415,702	451,932	41,570
1,100	482,074	547,224	48,207
1,200	576,736	606,962	57,674
1,350	687,390	716,075	68,739

**Table 3:** Decision variables and number of possible solutions for the target water supply network.

Target water supply network	Number of water supply network decision variables in the initial design	Total length of the pipeline	Number of possible solutions
Six zones of Saemangeum (loop type)	356	40,440 m	18^356^ ≒ *∞*

**Table 4:** Cost data based on pipe diameters as applied to Saemangeum.

Control parameters	Set value
HMS	30
HMCR	0.97
PAR	0.01
Constraint condition (pressure, *h*)	10 < *h* < 35
Constraint condition (pipe velocity, *v*)	0.01 < *v* < 2.5

**Table 5:** Comparisons of the costs incurred upon applying the optimal design versus the initial design.

Target water supply network	Initial design cost (**₩**)	Optimal design cost (**₩**)	Variation (%)
Six zones of Saemangeum (looped type)	11,200,114,720	10,182,733,295	−9.08

**Table 6:** Analysis results of the optimal and initial hydraulic designs (based on statistical values of the nodal head and pipe velocity).

Target water supply network	Nodal pressure head (m)				Pipe velocity (m/s)			
	Min.	Max.	Avg.	Var.	Min.	Max.	Avg.	Var.
Six zones of Saemangeum (looped type)	17.65	31.66	23.04	13.95	0.01	1.92	0.97	0.16
Optimal design	10.00	29.08	15.36	23.68	0.02	2.46	1.18	0.29

**Table 7:** Optimal design results and cost comparison of the initial plan (three cases).

Target water supply network	Initial design costs (**₩**)	Optimal design costs (**₩**)	Variation (%)
Loop type (plan 1)	11,200,114,720	10,182,733,295	−9.08
Branch type (plan 2)	10,484,719,750	10,044,962,405	−4.19
Pump type (plan 3)	11,503,515,255	11,586,379,380	+0.72

**Table 8:** Analysis results of the optimal and initial hydraulic designs (three cases).

Target water supply network		Nodal pressure head (m)				Pipe velocity (m/s)			
		Min.	Max.	Avg.	Var.	Min.	Max.	Avg.	Var.
Loop type (plan 1)	Initial plan	17.65	31.66	23.04	13.95	0.01	1.92	0.97	0.16
	Optimal design	10.00	29.08	15.36	23.68	0.02	2.46	1.18	0.29

Branch type (plan 2)	Initial plan	10.45	31.66	21.24	23.55	0.09	2.22	1.11	0.09
	Optimal design	10.00	29.08	14.28	20.63	0.15	2.40	1.08	0.30

Pump type (plan 3)	Initial plan	0.5	30.79	25.17	10.63	0.07	1.89	0.95	0.06
	Optimal design	10.00	30.79	16.14	29.26	0.22	2.49	1.36	0.25
